# Heterozygote of TAP1 Codon637 decreases susceptibility to HPV infection but increases susceptibility to esophageal cancer among the Kazakh populations

**DOI:** 10.1186/s13046-015-0185-y

**Published:** 2015-07-25

**Authors:** Ningjing Zou, Lan Yang, Ling Chen, Tingting Li, Tingting Jin, Hao Peng, Shumao Zhang, Dandan Wang, Ranran Li, Chunxia Liu, Jinfang Jiang, Lianghai Wang, Weihua Liang, Jianming Hu, Shugang Li, Chuanyue Wu, Xiaobin Cui, Yunzhao Chen, Feng Li

**Affiliations:** Department of Pathology and Key Laboratory for Xinjiang Endemic and Ethnic Diseases, Shihezi University School of Medicine, North 4th Road, Shihezi, Xinjiang 832002 China; Department of Oncology, Tongji Hospital, Huazhong University of Science and Technology, Wuhan, 430030 China; Department of Pathology, University of Pittsburgh, Pittsburgh, PA 15261 USA

**Keywords:** Esophageal squamous cell carcinoma, Human papillomavirus, Transporter associated with antigen processing, Single nucleotide polymorphism

## Abstract

**Background:**

The role of human papillomavirus (HPV) may be involved in the development of esophageal cancer (EC) and the polymorphic immune response gene transporter associated with antigen processing (TAP) may be involved in HPV persistence and subsequent cancer carcinogenesis. The current study aims to provide association evidence for HPV with EC, to investigate TAP1 polymorphisms in EC and assess its association with HPV statuses and EC in Kazakhs.

**Methods:**

The HPV genotypes in 361 patients with EC and 66 controls selected from Kazakh population were evaluated using PCR. Polymerase chain reaction-restriction fragment length polymorphism (PCR-RFLP) was performed to detect two SNPs of TAP1 in 150 cases comprised of 75 HPV^+^ and 75 HPV^-^ patients and 283 pure ethnic population of Kazakh and evaluate their associations with susceptibility to EC. A case-to-case comparison based on the genotyping results was conducted to address the function of TAP1 variants in the involvement of HPV.

**Results:**

The presence of four HPV genotypes in EC tissues ― including HPV 16, 18, 31, 45 ― was significantly higher at 64.6 % than those in controls at 18.2 % (*P* < 0.001). Such presence was strongly associated with increased risk of EC (OR 8.196; 95 % CI 4.280–15.964). The infection of HPV16, and multi-infection of 16 and 18 significantly increase the risk for developing EC (OR 4.616, 95 % CI 2.099–10.151; and OR 6.029, 95 % CI 1.395–26.057 respectively). Heterozygote of TAP1 D637G had a significantly higher risk for developing EC (OR 1.626; 95 % CI 1.080–2.449). The odds ratio for HPV infection was significantly lower among carriers of TAP1 D637G polymorphism (OR 0.281; 95 % CI 0.144–0.551).

**Conclusions:**

HPV infection exhibits a strong positive association with the risk of EC in Kazakhs. Heterozygote of TAP1 D637G decreases susceptibility to HPV infection in patients with EC but increases susceptibility to EC among the Kazakh populations.

## Introduction

Esophageal cancer, one of the most aggressive malignancies originating in the gastrointestinal tract, as well as, the sixth most frequent cause of cancer-related death worldwide, with wide geographic, ethnic or social cultural variation, results in more than 400,000 deaths annually [1, 2]. The two main histological types of esophageal cancer: esophageal squamous cell carcinoma (ESCC) and esophageal adenocarcinoma (EAC), of which the former is predominant type accounting for more than 80 % in China [3]. Comparing with other areas and ethnic groups of China, the Kazakh population, a nomadic tribe and mainly residing in the northwest of Xinjiang Province, shows high incidence and mortality of ESCC, with the highest age-adjusted mortality of 90.7/100,000 among all of the ethnic populations in Xinjiang [4]. Epidemiological studies have shown that heavy smoking and alcohol consumption, the main environmental risk factors for ESCC in Europe and North America [5–7], however seem to be minor risk factors for ESCC in the high-incidence areas in China [8, 9]. Therefore, other risk factors should be considered to explain the high incidence of ESCC in these areas.

Human papillomavirus (HPV), a nonenveloped double-stranded DNA virus with more than 120 kinds of subtypes have been separated, including more than 80 kinds of DNA sequencing, is a crucial tumor-related virus. HPV DNA has been detected in more than 90 % of cervical cancer specimens [10]. Notably, several studies indicated that HPV infection was found in extra genital cancers, such as 30 % of the human head and neck cancer and oral and oropharyngeal squamous cell carcinoma [11, 12], however, the etiological implication of HPV in these malignancies remains controversial [13]. In 1982, the association between HPV and EC was first reported that HPV infection caused pathological lesions in EC specimens [14]. Subsequent studies conducted in different geographic areas and ethnic groups, such as Han ethnic group in eastern China (Linxian and Anyang in Henan Province, Cixian in Hebei, Yangcheng in Shanxi and the northern Jiangsu province) and Kazakh minority residing in the northwest part of China, have shown large variations in the prevalence of HPV among EC patients, ranging from 0 to 100 % [15–20], which brought about opposite conclusions in the influence of HPV on the development of EC. The discrepancies may be generally attributed to the small size of the samples, differences in immunological and molecular methods, as well as inter-laboratory variability in sample collection and handling [21, 22]. In previous studies, HPV infection rate in EC in Kazakh patients varied from 18.6 to 41.1 % [19, 23, 24]. It comes up that the role of HPV infection in esophageal epithelium carcinogenesis is puzzling.

However, HPV alone is not sufficient to induce malignant transformation. The long-held theory of immune surveillance indicates that the alert immune system permanently prevent the growth of cancer by recognizing and eliminating the vast majority of incipient cancer cells and thus nascent tumors [25]. The major histocompatibility complex (MHC) plays a major role in the immune response against viral infections and transformed cells by presenting peptide antigens to cytotoxic T lymphocytes [26–28]. The transporter associated with antigen processing (TAP), whose genes are encoded in the MHC class II region of chromosome 6, is a critical component of the major histocompatibility complex (MHC) class I antigen presentation [29, 30]. Transporter associated with antigen processing is composed of two integral membrane proteins, TAP1 and TAP2, TAP1 functions by providing a supply of candidate peptides to the MHC-I molecules within the peptide loading complex and by transporting antigen peptides from the cytoplasm into the endoplasmic reticulum, [31, 32]. Regarding EC, the loss of expression of MHC class I and TAP1 has been reported to render some tumor cells to escape the immune surveillance and contribute to the clinical course of EC [33].

Some single nucleotide polymorphisms (SNPs) of TAP gene appear to influence the antigen peptide selection and transport process, and have been confirmed to be susceptible to various human diseases including immune diseases [34–36], infectious diseases [37–40] and tumors [41–43]. However, there has been only one study on the association of TAP polymorphisms and EC [44]. In this study, five gene polymorphisms including two at TAP1 and three at TAP2 were genotyped using polymerase chain reaction-restriction fragment length polymorphism (PCR-RFLP) method in 265 cases of ESCC and 357 controls. The results show no significant difference between the polymorphisms at TAP1 I333V and D637G and EC in Anyang, Henan. Given that TAP1 plays an important role in immune response and the polymorphisms of this gene may result in the conformational and functional change, and thus may affect individual’s susceptibility to HPV infection and subsequently the HPV-associated cancer development. What’s more, the distribution of Codon 333 and 637 polymorphisms of TAP1 and its relationship with HPV infection and EC remains unknown among the Kazakh population. Therefore the two potentially functional SNPs of this immune response gene to HPV must be investigated to define individuals with higher risk of developing malignant disease.

Therefore, the present study is employed to identify whether HPV infection is linked to the etiology of EC using PCR in 316 EC specimens and 66 biopsy samples of normal esophageal squamous epithelium from the Yili, an area with high incidence of EC. Additionally, 150 paraffin-embeded tumor specimens and 283 matched cancer-free controls were used to investigate the association between TAP1 polymorphisms and risk of EC in this ethnic and to accumulate evidence regarding the potential role of TAP polymorphisms in the disease. Subsequently, a case-to-case comparison based on the genotyping results was conducted to address the possible function of the TAP1 variants in the involvement of HPV in Kazakh patients with EC.

## Materials and methods

### Specimens used for detecting HPV infection

A total of 316 EC specimens of Kazakh in formalin-fixed paraffin-embedded archival tissues were collected from the People’s Hospital of Xinjiang Uyghur Autonomous Region, the First University Hospital, Shihezi University School of Medicine and several hospitals in the Yili Kazakh Autonomous Prefecture. The 316 cases were diagnosed between January 1990 and March 2014, and they were all diagnosed as ESCC. The pathological grade of tumors was classified at the time of diagnosis. Patients had received neither chemotherapy nor radiotherapy before endoscopies and surgery. All samples were surgically resected and fixed in 10 % buffered-formalin, routinely processed, and paraffin-embedded. The specimens of Kazakh patients were all obtained from the high-incidence areas in Xinjiang. From the patients’ medical records, we gathered data on clinical pathological variables such as tumor site, invasion depth, and distant metastasis. All cases of pathological diagnosis for the tumor-node- metastasis (TNM) stages were evaluated according to Cancer Stage Manual 7th Edition 2009 issued in 2009 by the American Joint Committee on Cancer (AJCC/UICC). Informed consent of each patient was obtained, and study protocol was approved by the Institutional Review Board at Shihezi University School of Medicine. Biopsy samples of normal esophageal squamous epithelium were available from 66 control patients matched for ethnicity, who were from Kazakh-gathering place in Xinyuan, Yili and participated in the project of Early Diagnosis and Treatment of EC in Xinjiang Province. Each control had undergone upper gastrointestinal endoscopy and the esophageal tissue samples were confirmed as histologically normal. At the same time, the control subjects’ corresponding pathological and clinical data were gained.

The patients in this study were comprised of 187 men and 129 women with an average age of 52.89 ± 8.923 years (ranging from 23 to 79 years). In addition, a total of 36 men and 30 women control subjects were matched with an average age of 50.67 ± 11.542 years (ranging from 19 to 64 years). The difference between cases and controls is of no significance in gender (*P* = 0.488) and age (t = 1.741, *P* = 0.083) distribution. The cases included 106 (33.5 %) well-differentiated patients (group G1), 162 (51.3 %) moderately differentiated patients (G2), and 48 (15.2 %) poorly differentiated patients (G3). When evaluated according to Cancer Stage Manual 7th Edition 2009, the 316 ESCC cases, 48 (15.2 %) were classified as stage I, 183 (58.0 %) as stage II, 75 (23.7 %) as stage III, and 10 (3.1 %) as stage IV. Of the 316 ESCC cases, 30 (9.5 %) were classified as T1, 158 (50.0 %) as T2, 123 (38.9 %) as T3, and 5 (1.6 %) as T4.

### Specimens used for testing TAP1 gene polymorphisms

The 316 Kazakh patients with EC mentioned above, which had been tested for HPV infection, was then divided into two groups (204 cases infected with HPV and 112 cases that weren’t), from both of which we selected 75 cases respectively and at random, so a total of 150 paraffin-embedded tumor specimens were used for genotyping TAP1 gene. The 283 controls were selected randomly from a pool of cancer-free subjects recruited from Kazakh ethnic population in Xinyuan, Yili, who had visited the hospital for a conventional cancer screening program. Blood samples were collected and DNA was extracted for the genotyping of TAP1 gene. Informed consent was obtained from all participants in this study.

The patients in this study were comprised of 94 men and 56 women with an average age of 52.29 ± 9.571 years (ranging from 23 to 75 years). In addition, a total of 154 men and 129 women control subjects were matched with an average age of 50.25 ± 11.991 years (ranging from 21 to 72 years). The difference in gender distribution between cases and controls is of no significance (*P* = 0.098), however, age distribution differs significantly between cases and controls (t = 1.801, *P* = 0.072).

### Detection of HPV DNA

**Fig. 1:**
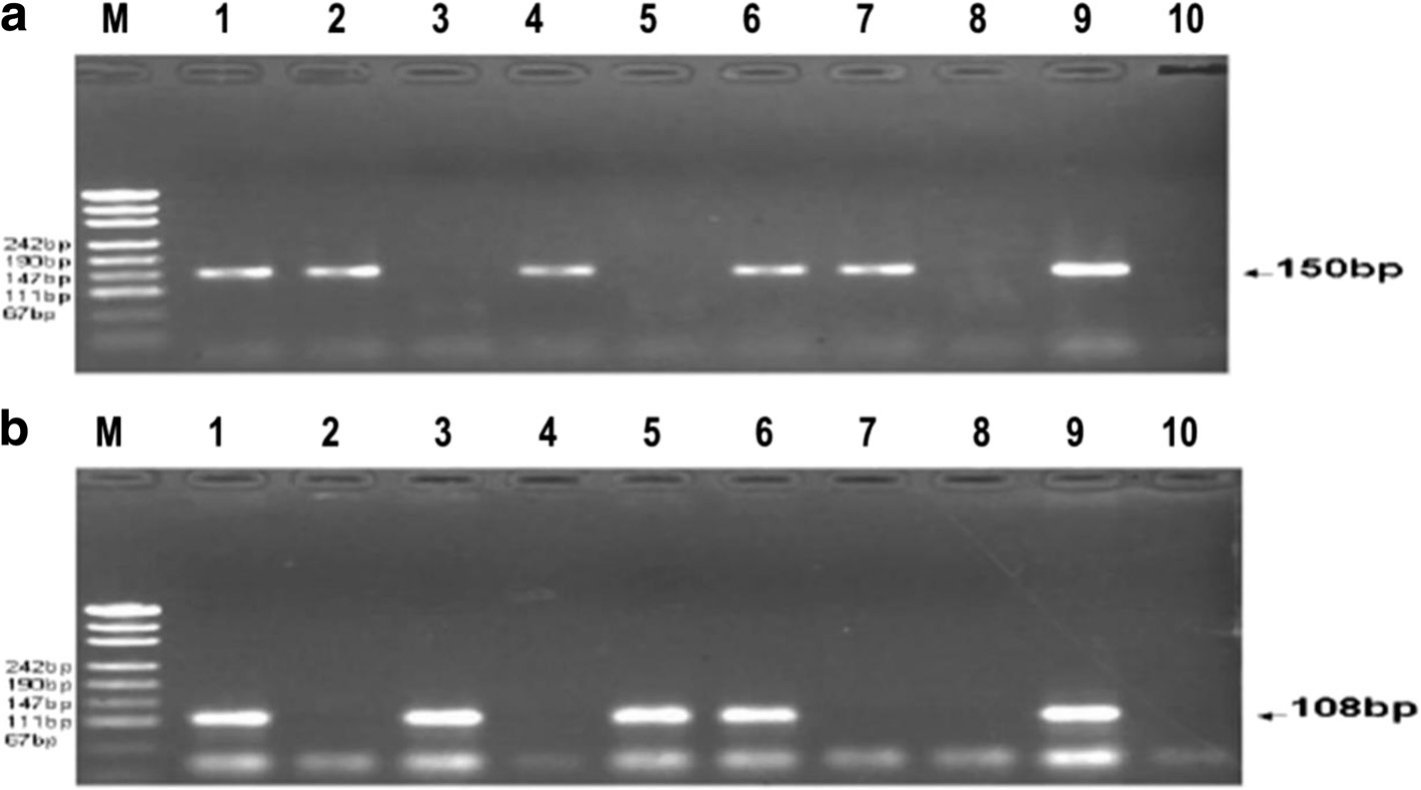
Electrophoretic analysis of the HPV genotyping using PCR. (**a**) GP5^+^/GP6^+^ PCR. Lane 1, 2, 4, 6 and 7 in HPV infection (150 bp); Lane 3 and 4 without HPV infection; Lane 8, negative control; Lane 9, positive control; Lane 10, blank control; M, molecular weight marker. (**b**) Human papillomavirus PCR results. Lane 1, 3, 5 and 6 in HPV16 infection (150 bp); Lane 2, 4 and 7 without HPV16 infection; Lane 8, negative control; Lane 9, positive control; Lane 10, blank control; M, molecular weight marker

**Fig. 2:**
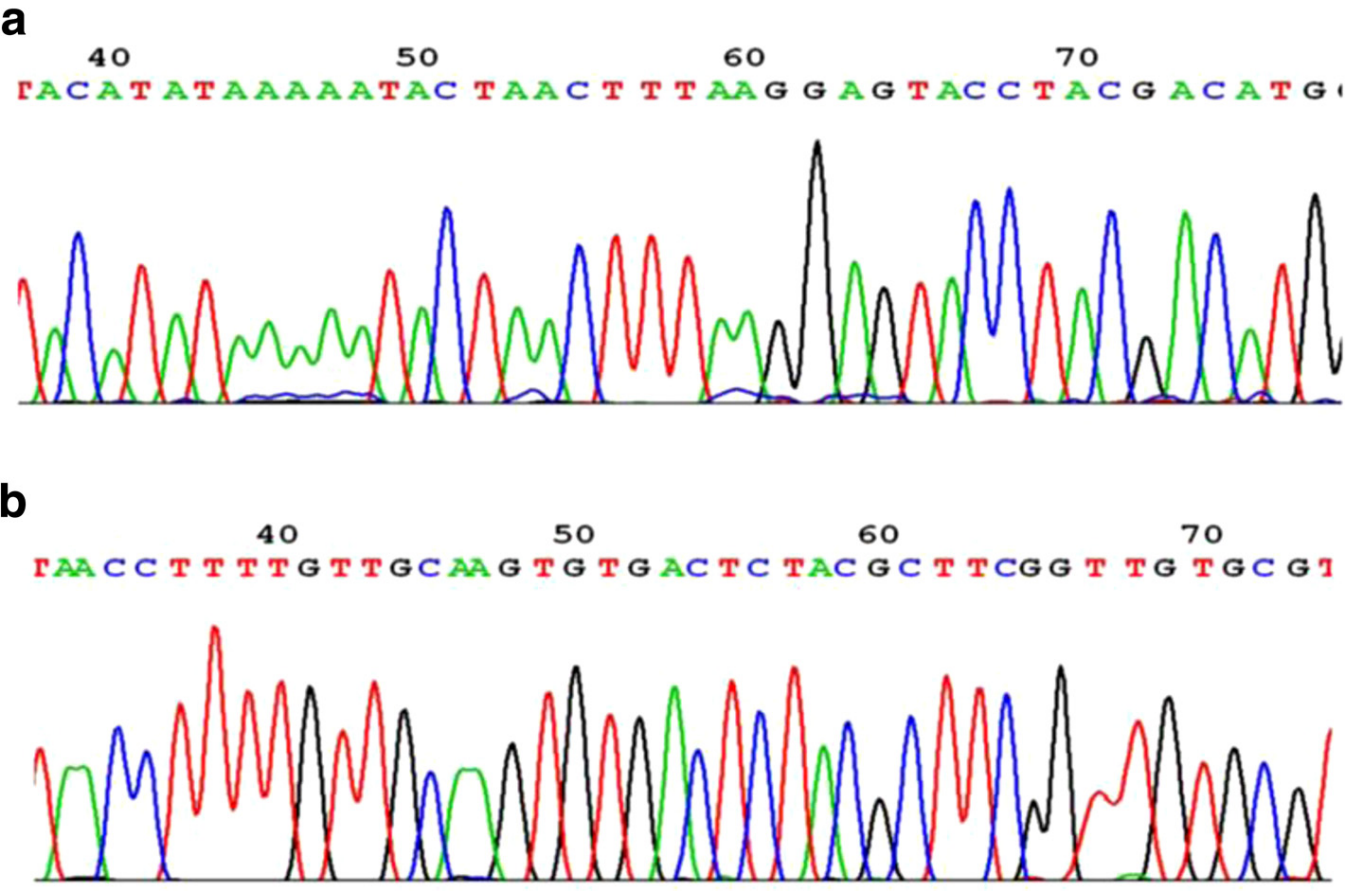
Sequencing map of the genotype for the HPV L1 (**a**) and HPV 16E7 (**b**)

DNA was extracted from the paraffin sections and biopsy samples using standard proteinase K digestion and a tissue DNA extraction kit (Qiagen, Hilden, Germany) according to the manufacturer’s instructions. As an internal control, all purified genomic DNA samples were successfully tested by polymerase chain reaction (PCR) with a human β-actin primer set (forward: 5′-CAGACACCATG GTGCACCTGAC-3′ and reverse: 5′-CCAATAGGCAGAGAGAGTCAGTG-3′), indicating that the quality and quantity of DNA were suitable for detecting the presence of HPV. The HPV infection was first determined by PCR using non-degenerate primer set, GP 5+/6+, Forward primer: TTGGATCCTTTGTACTGTGGTAGATACTAC and Reverse primer: TTGGATCCGAAAAATAAACTGTAAATCATATTC, which produce a 150 bp ragment of L1 gene in a wide range of HPV types. For HPV 16 DNA detection, E7 was amplified with forward primer GATGAAATAGATGGTCC AGC and reverse primer GCTTTGTACGCACAACCGAGC. For each PCR reaction, 5 μL extracted concentrated DNA was used in a final reaction volume of 25 μL. The reaction was initially carried out at 95 °C for 10 min, followed by 40 cycles of denaturation at 94 °C for 30 s, annealing at 42 °C for 90 s, and extension at 72 °C for 30 s, with a final extension at 72 °C for 5 min. The amplicons were then denatured and subjected to hybridization. Assays of the samples were run in triplicate, with positive and negative controls (CaSki cell DNA generously provided by Professor Yang Ke, Beijing Institute for Cancer Prevention Laboratory and distilled water) (Fig. 1). β- actin was used as a DNA control. To confirm the accuracy of the HPV genotyping by PCR, 10 % DNA sequence of the positive products was then identified by NCBI Blast (Fig. 2). A 100 % match was identified between the results of DNA sequence displayed in the Genbank and HPV genotype detected.

### Genotyping TAP1 I333V and D637G polymorphisms

**Fig. 3:**
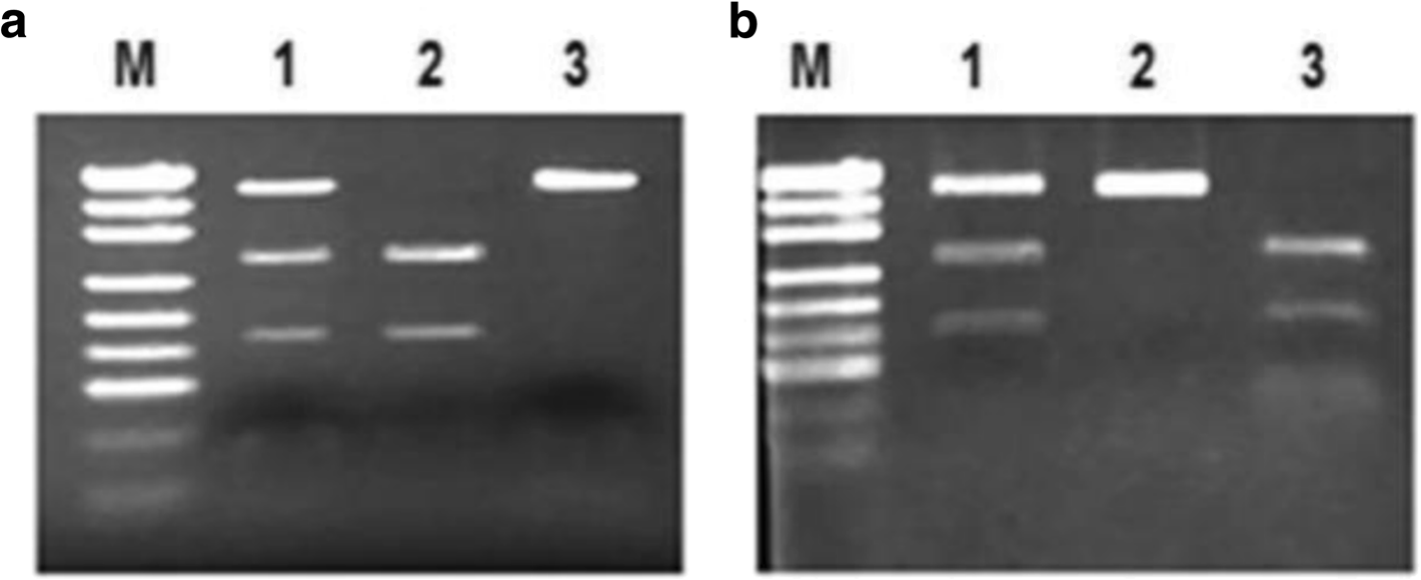
PCR-RFLP assay for analyzing the TAP1 Codon333 and 637 polymorphisms. PCR product was digested by restriction enzyme and visualized in 3 % agarose gel stained with ethidium bromide. (**a**) The typical genotypes of TAP1 Codon333 A/G. Lane 1: AG genotype (430,274 and 156 bp); lane 2: AA genotype (274 and 156 bp); lane 3: GG genotype (430 bp). (**b**) The typical genotypes of TAP1 Codon637 A/G. Lane 1: AG genotype (405,260 and 145 bp); lane 2: GG genotype (405 bp); lane 3: AA genotype (260 and 145 bp). M, molecular weight marker

**Fig. 4:**
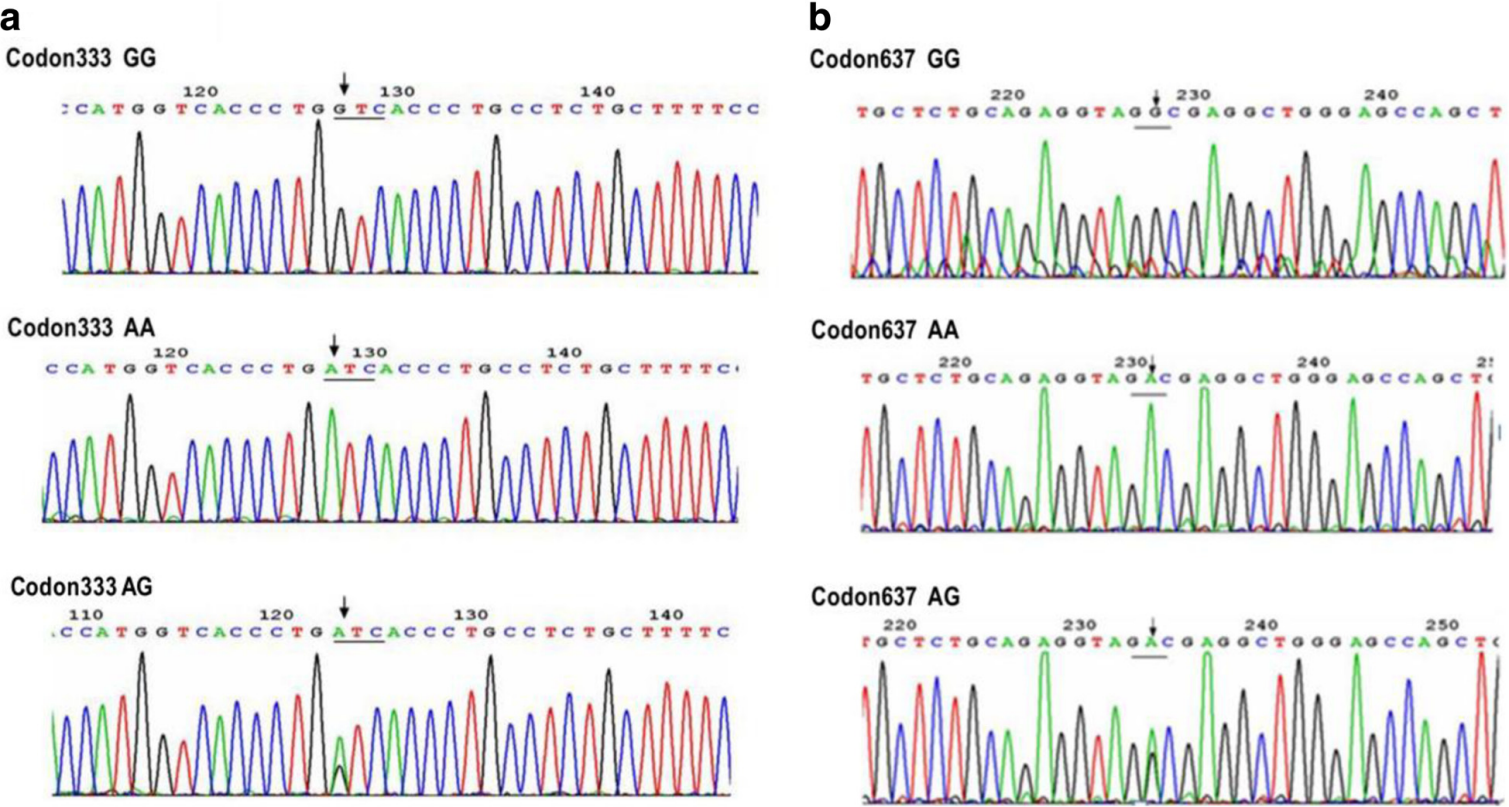
Sequencing map of the genotype for the TAP1 Codon333 polymorphism (**a**) and TAP1 Codon637 polymorphism (**b**)

DNA was extracted from the paraffin sections and blood samples using standard proteinase K digestion and a tissue DNA extraction kit (Qiagen, Hilden, Germany) according to the manufacturer’s instructions. The two previously documented polymorphisms in TAP1 coding regions are previously documented. The primers used in PCR to detect for Codon 333 were as follows: forward primer GCAGGTAACATC ATGTCTCG and Reverse primer: GACAGATTGTGGGGAGAAGC, and for Codon 637 were as follows: forward primer CAGTAGTCTTGCCTTTATCC and Reverse primer: ATGACTGCCTCACCTGT AAC. The PCR was carried out in 25 ml reaction mixture containing genomic 1 ml DNA, 2.5 ml 10 × PCR buffer (100 mM Tris, pH 7.8, 100 mM NaCl, 10 mM EDTA and 0.5 % SDS), 1.8 ml of 25 mM MgCl_2_, 0.5 ml of 10 mM dNTP each, 10 pmol primer and 1.5 U of Taq polymerase (Promega, Madison, WI, USA). The reaction was initially carried out at 94 °C for 2 min, followed by 35 rounds of thermal cycling were in the conditions: denaturation at 94 °C for 40 s, annealing at 57.5 °C for 40s, extension at 72 °C for 40 s and the final extension was carried out at 72 °C for 10 min. The terminator ready sequencin was performed in a total volume of 20 ml containing 8 ml of PCR product, 2.5 ml 10 × PCR buffer, the enzymes used were 7 U of Bcl1 and Acc1 for genotyping Codon 333 and Codon 637 respectively. Cycling conditions were as follows: initial denaturing 96 °C for 2 min, followed by 30 cycles at 94 °C for 10 s, 50 °C for 5 s and 60 °C for 4 min. Restriction enzyme digestion with Bcl1 and Acc1 (Promega, Madison, WI, USA) of the PCR product was carried out overnight and analyzed on a 3 % agarose gel. DNA products were visualized by ethidium bromide staining (Fig. 3). The −333A and −637A showed two fragments (homozygous for the allele −333A and −637A), while its homologue −333G and −637G was undigested and resulted in a single band (homozygous for allele −333G and −637G). The presence of all three fragments defined heterozygotic individuals. To confirm the accuracy of TAP1 I333V and D637G genotyping by PCR, 10 % DNA sequence of the PCR products was then identified by NCBI Blast (Fig. 4). A 100 % match was identified between the results of DNA sequence displayed in the Genbank and HPV genotype detected.

### Statistical analysis

All statistical analyses were performed using SPSS version 17.0, U.S. Hardy-Weinberg equilibrium in controls was tested by chi-square tests comparing observed and expected genotype frequencies. The presence of HPV DNA in case–control study, Clinic pathological characteristics of HPV-positive and HPV-negative patients with ESCC, and the differences in the distributions of genotype of TAP1 I333V and D637G were analyzed by chi-square test. Logistic regression models for genotype were used to examine TAP1 I333V and D637G polymorphisms with risk of Kazakh EC, the effects of TAP1 D637G on risk of ESCC with different tumor depth, histological grade and clinical stage, and the association of HPV infection with TAP1 I333V and D637G polymorphisms in Kazakh EC. Results were given as P values, odds ratios (ORs) and 95 % confidence intervals (95 % CIs). All statistical analyses were two-sided, and *P* < 0.05 was considered statistically significant.

## Results

### Prevalence of HPV Infection in Kazakh EC Patients

Polymerase chain reaction (PCR) was employed to screen HPV infection statuses in 361 patients with EC and 66 controls, and the results are shown in Table 1. The prevalence of HPV DNA was higher in EC cases than that in the control group (64.6 % vs. 18.2 %, *P* < 0.001). Four HPV genotypes were identified in both EC tissue and normal esophageal tissue, including HPV16, 18, 31 and 45. HPV DNA was identified in 204 of 316 tumor tissue samples. There are totally 56 EC patients and 3 normal controls with HPV multi-infection. Using the unconditional logistic regression models, the association between HPV infection and Kazakh patients with EC was evaluated. Apart from HPV45, the HPV detected, including HPV16 (OR, 3.941;95 % CI, 1.926–7.955), HPV18 (OR, 3.390;95 % CI, 1.411–8.145) and HPV31 (OR, 3.487; 95 % CI, 1.050–11.582), exhibited a strong positive association with the risk of EC in Kazakhs. A higher OR value (OR, 8.196; 95 % CI, 4.280–15.964) was observed in patients who are tested positive for HPV DNA (Table 1).

**Table 1:** Analysis of the association between HPV infection and Kazakh patients with esophageal carcinoma

Types	Case (*n* = 361)	Control (*n* = 66)	*χ* ^2^	OR (95 % CI)
	n (%)^a^	n (%)^a^		
HPV L1	204 (64.6)	12 (18.2)	47.786***	8.196 (4.28–15.964)
HPV16E7	130(41.1)	10 (15.2)	15.881***	3.941 (1.926–7.955)
HPV18E7	80 (25.3)	6 (9.1)	8.240**	3.390 (1.411–8.145)
HPV31E7	45(14.2)	3 (4.5)	4.671*	3.487 (1.050–11.582)
HPV45E7	23(7.3)	1 (1.5)	3.080	5.102 (0.677–38.466)
Multiple infection	56(17.7)	3 (4.5)	7.258**	4.523 (1.371–14.922)

“*”represents *P* < 0.05, “**”indicates *P* < 0.01, “***”depicts *P* < 0.001

^a^Because of the multiple infection, the total HPV-positive rate was smaller than the sum of the positive rates of each subtype

Since the high-risk HPVs, especially types 16 and 18, have been identified as the risk factor for cervical cancer as well as other cancers, to study further, HPV16 or/and 18 infected individuals were referred as the infected subjects and then, the association between risk of EC with HPV16 and 18 infection was assessed. The infection of HPV16, 18 and multi-infection of the two types significantly increase the risk for developing EC (OR 4.616, 95 % CI 2.099–10.151; OR 4.522,95 % CI. 1.553–13.165 and OR 6.029, 95 % CI 1.395–26.057, respectively) in this area (Table 2).

**Table 2:** Association of HPV 16 or/and 18 infection with risk of EC

Types		Case (*n* = 361)	Control (*n* = 66)	*χ* ^2^	OR (95 % CI)
HPV16E7	HPV18E7	n (%)	n (%)		
Negative	Negative	138 (43.7)	52 (78.8)		1.000
Positive	Negative	98 (31.0)	8 (12.1)	16.541^***^	4.616 (2.099–10.151)
Negative	Positive	48 (15.2)	4 (6.0)	10.063**	4.522 (1.553–13.166)
Positive	Positive	32 (10.1)	2 (3.0)	7.277**	6.029 (1.395–26.057)

“*”represents *P* < 0.05, “**”indicates *P* < 0.01, “***”depicts *P* < 0.001

### Association between HPV Infection and Clinical pathologic Characteristics of Patients with EC

To identify whether the HPV infection influences the clinical pathologic characteristics in Kazakh patients with EC, the association of HPV infection with nine characteristics including age, gender, smoking, drinking, tumor location, histological grade, depth of invasion, lymph node metastasis and clinic stage was examined. Our results show that no significant difference in the nine investigated clinical pathologic characteristics is indicated between the EC patients with HPV and patients without (Table 3).

**Table 3:** Clinic pathological characteristics of HPV-positive and HPV-negative patients with ESCC

Characteristics	Number	HPV^**+**^ Patients *n* = 204	HPV^**−**^ Patients *n* = 112	*χ* ^2^	P value
		n (%)	n (%)		
Age (years), mean ± SD	316	53.01 ± 8.202	52.67 ± 10.141		0.746^a^
Age (years)				0.301	0,584
<55	173	114 (65.9)	59 (34.1)		
≥55	113	90 (62.9)	53 (37.1)		
Gender				2.692	0,101
Male	187	127 (67.9)	60 (32.1)		
Female	129	76 (58.9)	53 (41.1)		
Smoking				0.081	0.775
Yes	82	54 (65.9)	28 (34.1)		
No	234	150 (64.1)	84 (35.9)		
Drinking				2.149	0.143
Yes	65	47 (72.3)	18 (27.7)		
No	251	157 (62.5)	94 (37.5)		
Location of the tumor				1.311	0.519
The upper thoracic portion	19	10 (52.6)	9 (47.4)		
The mid-thoracic portion	170	112 (65.9)	58 (34.1)		
The lower thoracic portion	127	82 (64.6)	45 (35.4)		
Histologic grade				0.331	0.848
Well-differentiated (G_1_)	106	67 (63.2)	39 (36.8)		
Moderately differentiated (G_2_)	162	107 (66.0)	55 (34.0)		
Poorly differentiated (G_3_)	48	30 (62.5)	18 (37.5)		
Depth of invasion				0320	0.572
T1/T2	188	119 (63.3)	69 (36.7)		
T3/T4	128	85 (66.4)	43 (33.6)		
Lymph node metastasis				1.127	0.288
No	193	129 (66.8)	64 (33.2)		
Yes	123	75 (61.0)	48 (39.0)		
Clinical stage				1.847	0.174
stage I/II	231	144 (62.3)	87 (37.7)		
stage III/IV	85	60 (70.6)	25 (29.4)		

^a^Student’s test

### TAP1 gene polymorphisms are predictors of EC risk in Kazakh populations

To evaluate the role of TAP1 gene polymorphisms in the MHC class II region in the association of Kazakh EC, two potentially functional SNPs in TAP1 were tested. The distribution of genotypes and alleles is significantly different between cases and controls at TAP1 D637G (*P* = 0.040 and *P* = 0.018 respectively), but not at TAP1 I333V (*P* = 0.250 and *P* = 0.118 respectively). TAP1 D637G allele (G) is significantly more common in patients than that in controls (*P* = 0.018), suggesting that the G allele is associated with EC in Kazakh population. Logistic regression models for genotype were derived to examine the association between TAP1 polymorphisms and the risk of Kazakh EC. Only TAP1 D637G polymorphism was found to be associated with cancer risk, which was significantly increased for carriers of at least one G allele (OR, 1.659; 95 % CI, 1.112–2.474) and heterozygote carriers of the variant allele G (OR, 1.626; 95 % CI, 1.080–2.449) (Table 4).

**Table 4:** Association of TAP1 gene polymorphisms with risk of Kazakh ESCC

Genotype or allele	Case n (%)	Control n (%)	P value	OR (95 % CI)
TAP1 D637G				
AA^a^	73 (48.7)	173 (61.1)		1.000 (reference)
AG	70 (46.7)	102 (36.1)	0.019	1.626 (1.080–2.449)
GG	7 (4.7)	8 (2.8)	0.246^b^	2.074 (0.725–5.930)
Combined variant genotypes				
AG + GG	77 (51.3)	110 (38.9)	0.013	1.659 (1.112–2.474)
A^a^	216 (72.0)	448 (79.2)		1.000 (reference)
G	84 (28.0)	118 (20.8)	0.018	1.477 (1.068–2.040)
TAP1 I333V				
AA^a^	82 (54.7)	134 (47.3)		1.000 (reference)
AG	62 (41.3)	130 (46.0)	0.232	0.780 (0.518–1.173)
GG	6 (4.0)	19 (6.7)	0.170	0.516 (0.198–1.345)
Combined variant genotypes				
AG + GG	68 (45.3)	149 (52.7)	0.112	0.724 (0.486–1.079)
A^a^	226 (72.0)	398 (70.3)		1.000 (reference)
G	74 (24.7)	168 (29.7)	0.118	0.776 (0.564–1.067)

^a^Reference group

^b^Fisher’s exact test

### Correlations of clinical pathological parameters and TAP1 D637G polymorphism in Kazakh patients with ESCC

To investigate the contribution of confounding factors such as gender and age to the risk for Kazakh ESCC, stratification analyses were conducted to evaluate the potential association of genetic variants of the TAP1 D637G polymorphism with risk of subgroup populations (Table 5). Interestingly, the results showed that the risk effects of AG/GG genotypes were more evident in female subjects (OR = 2.093). No significant association was found between TAP1 D637G polymorphism and ESCC with respect to age (*P* > 0.05).

**Table 5:** Correlations of clinicopathological parameters and TAP1 D637G polymorphism in Kazakh patients with ESCC

Parameters	AA	AG/GG	OR (95 % CI)
	(Cases/controls)	(Cases/controls)	
Gender^a^			
Male	48/92	46/62	1.422 (0.848–2.385)
Female	25/81	31/48	2.093 (1.107–3.954)*
Age^a^			
≤52	30/91	30/60	1.517 (0.831–2.769)
>52	43/82	37/50	1.411 (0804–2.478)
Tumor depth^b^			
T1/T2	37/173	48/110	2.040 (1.249–3.333)**
T3/T4	36/173	29/110	1.267 (0.735–2.184)
Histologic grade (G)^b^			
G1	27/173	27/110	1.573 (0.877–2.822)
G2	38/173	40/110	1.656 (0.999–2.741)
G3	8/173	10/110	1.966 (0.753–5134)
Clinical stage^b^			
I/II	53/173	58/110	1.721 (1.106–2.679)*
III/IV	20/173	19/110	1.494 (0.763–2.925)

We consider the common homozygotes of TAP1 as ORs of 1.000 for the reference genotype

“*”represents *P* < 0.05, “**”indicates *P* < 0.01, “***”depicts *P* < 0.001

^a^Stratification analysis to evaluate the effects of variant genotypes on the risk of ESCC by age and sex

^b^Logistic regression analysis for the effects of TAP1 variants on risk of ESCC with different tumor depth, histologic grade and clinical stage through logistic regression analyses. G1: well differentiated; G2: moderately differentiated; G3: poorly differentiated

The association between PLCE1 variants and histologic grade and clinical stage of ESCC was further evaluated (Table 5). When the ESCC patients were divided into 2 subgroups, T1/T2 and T3/T4, according to the AJCC TNM classification of carcinoma of esophagus, the carriers of AG/GG genotypes had a significantly increased risk for T1/T2 ESCC (*P* = 0.004, OR = 2.040). When the ESCC patients were divided into 2 subgroups, stage I/II and stage III/IV, the combined risk genotypes were found to be associated with stage I/II ESCC (*P* = 0.016, OR = 1.721).

### Relation of HPV positive of Kazakh patients with EC to TAP1 genotype

The results of genotype frequencies of the two investigated TAP1 gene polymorphisms in HPV-positive and HPV-negative patients are summarized in Table 6. The distribution of genotypes was significantly different between patients with HPV infection and those without at TAP1 D637G (*P* < 0.001), but not at TAP1 I333V (*P* = 0.056). To study further, we analyze the association between TAP1 D637G polymorphisms and HPV-associated Kazakh patients with EC. Differing from the wild-type AA homozygote, the combined AG/GG or AG variant genotypes were significantly associated with HPV-positive in EC patients (OR 0.255, 95 % CI 0.127–0.511; and OR 0.281, 95 % CI 0.144–0.551, respectively), although the homozygous mutant type of TAP D637G (GG) exhibited no association with HPV-positive EC patients (*P* = 0.691). We further evaluated the association between the risk of genotypes of TAP1 and risk of HPV-associated ESCC stratified by tumor depth. Interestingly, the results showed that differences between the protective effects of heterozygote and combined genotypes of TAP1 D637 were more significantly in T1/T2 subjects (*P* = 0.004, OR = 0.028 and *P* = 0.006, OR = 0.283, respectively) than in T3/T4 subjects (*P* = 0.020, OR = 0.286 and *P* = 0.026, OR = 0.311 respectively).

**Table 6:** Stratification analysis to evaluate the association of HPV positive patients with TAP1 polymorphisms in Kazakh ESCC by tumor depth

Parameters	TAP1 D637G (HPV^+^/HPV^-^ patients)		OR (95 % CI)	TAP1 I333V (HPV^+^/HPV^-^ patients)		OR (95 % CI)
	AA^a^	AG/GG		AA^a^	AG/GG	
Tumor depth						
T1/T2	21/16	13/35	0.283 (0.114–0.703)**	23/27	11/24	0.538 (0.218–1.330)
T3/T4	27/9	14/15	0.311 (0.109–0.888)*	21/11	20/13	0.806 (0.294–2.212)
T1-T4	48/25	48/25	0.281 (0.144–0.551)***	44/38	31/37	0.724 (0.380–1.380)

“*”represents *P* < 0.05, “**”indicates *P* < 0.01, “***”depicts *P* < 0.001

^a^Reference group. We consider the common homozygotes of TAP1 as ORs of 1.00 for the reference genotype

## Discussion

More than one hundred HPV subtypes have been described [45], over forty of which have been shown to be involved in human disease, and these subtypes are traditionally characterized by the sequence of their L1 gene. The involvement of HPV in etiology of EC has drawn interest in the past three decades since Syrjanen first found 40 % of EC patients had similar histological changes to the genital warts in reproductive tract in 1982 [14]. The majority of studies have identified HPV in ESCC samples, with infection rates ranging from 17.1 [46] to 78.11 % [44], detected via amplification of the L1 gene. One study reported an infection rate of 100 %, which was determined by detection of the HPV16 E6 and E7 genes in early cancer cases in a high-risk area using PCR and ISH in Anyang, Henan [47]. While a recent study in east China, in which 23 different HPV types was screened using a human papillomavirus genotyping kit and the P16^INK4a^ protein was detected using immunohistochemistry (ISH), shows the absence of human papillomavirus in EC [20]. These two extreme values may best illustrated that the incidence of HPV infection in EC varies from the high-incidence to the low-incidence areas [15, 17, 22, 47–51], which is not in accordance with HPV infection in cervical carcinoma. This finding suggests that HPV infection exerts a complex effect on carcinogenesis of the EC.

The PCR method was adopted to assess prevalence of four high-risk HPV in the 361 Kazakh patients with EC and 66 cancer-free controls obtained from several hospitals between 1990 and 2014 in the Kazakh Autonomous Prefecture of Xinjiang, an area with high incidence of EC located in northwestern China. The significantly high prevalence of HPV in the Kazakh patients with EC indicated that HPV infection significantly affect the carcinogenesis of the EC in Kazakh population (64.6 % vs. 18.2 %, *P* < 0.001). It has been shown that human papilloma virus is found frequently in ESCCs in high incidence areas. What’s more, the frequency of HPV infection in the tumors observed in the current study much higher than the average rate from a systematic review and a formal meta-analysis of the literature on HPV detection in 10,234 EC cases from 1954 to 2012 [52]. Additionally, our present study showed the incidence of HPV16 infection in Kazakh EC patients (41.1 %) was significantly higher than in the control group (15.2 %), and, it has been proposed that HPV16 infection may play a role in esophageal carcinogenesis. A similar observation was also revealed in previous studies in Xinjiang Kazakhs [23, 53]. Nevertheless, some studies demonstrate no relationship between HPV infection and EC, which may reflect discrepancy of different race, different ethnic and different region. In our study, strict quality controls on the object and method of the study was performed strictly. Therefore, we can confirm that HPV infection is one of the important factors in high incidence of Xinjiang ethnic Kazakh EC.

The data confirmed no significant association between the presence of HPV and the histological grade of EC. While in certain reports, well-differentiated tumors tended to have either HPV16 or HPV16/6 infection, suggesting that HPV can initiate the development of EC and explaining the higher survival rate indicated in HPV-positive patients with EC [13, 19]. Nevertheless, other study reported that a positive HPV16/18 rate and viral load were more often in poorly differentiated EC cases, although no significant correlation was confirmed [24]. The discrepant results in the literatures may be attributed to the difference in detection methods, sample size, and patient heterogeneity because of the variety of ethnicities in the sample although the samples cases were obtained from the same region. Thus, the conclusions concerning the influence of HPV on various differentiation of EC warrant confirmation.

Host factors such as the immune systems and genetic factors also seem to be important factors in HPV persistence and subsequent cancer carcinogenesis [52, 54]. The polymorphisms of TAP1 genes may result in the conformational and functional change of TAP, which can influence the antigen peptide selection and transport process [55]. There are several studies on the relationship between TAP (including TAP1 and TAP2) polymorphisms and various carcinoma, and only TAP1 polymorphisms have been confirmed to be susceptible to various tumor types. One study shows that no significant difference in genotype distribution of TAP1 and TAP2 polymorphisms was observed in women with cervical intraepithelial neoplasm (CIN) and controls [56], and a similar study conducted in India succeeded to reproduce this observation [57], however, in another similar study, significant differences in allele distribution between women with high-grade cervical neoplasm (CIN II or III) and women without was seen for both TAP1 I333V (*P* = 0.02) and TAP1 D637G (*P* = 0.01) [58]. In addition, G allele at TAP1 Codon 637 is associated with nasopharyngeal carcinoma (NPC) in Han population in Yunnan, China (OR, 1.88; 95 % CI, 1.35–2.82; *P* < 0.001), and EBV pathogenesis in NPC might be facilitated by polymorphisms in the TAP1 proteins [59].

The present case–control study has analyzed the association between TAP gene polymorphisms and the risk of EC in Kazakh population in Xinjiang, China. The important finding is that the heterozygote of TAP1 D637G (AG) genotypes were associated with a 1.626-fold higher risk for the development of EC, whereas the GG genotype was not associated with the risk of developing EC, suggesting that G allele at TAP1 D637G is a risk factor for EC, which is contrary to the study of Cao et al [44]. The reason for this discrepancy might be due to genetic heterogeneity between Kazakh and Han population, according to the theory that the Han Chinese population, though seeming homogeneous, exhibits a complicated substructure as the genetics of different Han Chinese populations differ greatly [60]. In the current samples, false-positive or false-negative associations owing to population substructure were less likely to exist, because the carefully ascertained, relatively homogeneous case–control samples of northwestern Kazakh belong to a single geographic location of the province of Xinjiang. This polymorphism has been shown to vary with ethnic and geographical distribution. However, its influence has not been elucidated in the Kazakh population.

Epidemiologic and molecular studies have shown that all virus agents and the genetic factors are involved in the initiation and progression of EC [17]. Another important finding in the present study is that HPV-positive rate is lower for patients carrying allele G at TAP1 D637G, and this provides an illustration to the theory that TAP facilitates the detection of HPV by MHC-I molecules and contributes to detection and eradication of HPV despite various immune evasion mechanisms of the virus [61].

## Conclusion

Taken together the present study demonstrated that G allele at TAP1 Codon637 decreases susceptibility to HPV infection in patients with EC among the Kazakh populations, however, it is associated with an increased risk of EC in this ethnic. This finding suggests that the polymorphism of TAP1 Codon637 exerts complicated influence on EC in Kazakh population. However, given that the mechanisms by which TAP1 polymorphisms may influence the course of HPV infections remains under investigation, further studies need to be conducted to elucidate the influence of TAP1 polymorphisms in HPV-associated EC in the Kazakh population.
